# High burden of Carbapenem-resistant Enterobacteriaceae (CRE) fecal carriage at a teaching hospital: cost-effectiveness of screening in low-resource setting

**DOI:** 10.1186/s13756-017-0200-5

**Published:** 2017-05-02

**Authors:** Abdul Rahman Zaidah, Nurul Izzah Mohammad, Siti Suraiya, Azian Harun

**Affiliations:** Department of Medical Microbiology & Parasitology, School of Medical Sciences, Universiti Sains Malaysia, 16150 Kubang Kerian, Kelantan Malaysia

**Keywords:** Carbapenem-resistant *Enterobacteriacea*, *Klebsiella pneumoniae*, Screening

## Abstract

**Background:**

Infections by multidrug-resistant gram-negative bacteria (MDR-GNB) have been continuously growing and pose challenge to health institution globally. Carbapenem-resistant *Enterobacteriacea* (CRE) was identified as one of the MDR-GNB which has limited treatment options and higher mortality compared to those of sensitive strains. We report an increased burden of CRE fecal carriage at a hospital in the North-eastern region of Malaysia.

**Methods:**

A retrospective descriptive study from August 2013 to December 2015 was conducted in the Medical Microbiology & Parasitology laboratory of Hospital Universiti Sains Malaysia, which is a tertiary teaching hospital with more than 700 beds. This hospital treats patients with various medical and surgical conditions. Suspected CRE from any clinical specimens received by the laboratory was identified and confirmed using standard protocols. Polymerase chain reaction (PCR) assay was performed to determine the genotype.

**Results:**

Altogether, 8306 Enterobacteriaceae was isolated from various clinical specimens during the study period and 477/8306 (5.74%) were CRE. Majority of the isolated CRE were *Klebsiella* [408/477, (85.5%)], of which *Klebsiella pneumoniae* was the predominant species, 388/408 (95%). CRE were mainly isolated from rectal swab (screening), 235/477 (49.3%); urine, 76/477 (15.9%); blood, 46/477 (9.6%) and about 7.1% from tracheal aspirate. One hundred and thirty-six isolates were subjected to genotype determination and., 112/136 (82.4%) showed positive detection of New Delhi metallo-β-lactamase 1 (NDM-1) gene (*bla*_NDM1_).

**Conclusion:**

The study noted a high numbers of CRE isolated especially from rectal swabs. Active screening results in significant cost pressures and therefore should be revisited and revised, especially in low resource settings.

## Background

Multidrug-resistant gram-negative bacteria (MDR-GNB) particularly Carbapenem-resistant *Enterobacteriaceae* (CRE) have become a threat to health institution globally [1, 2]. The Centers for Disease Control and Prevention (CDC) noted continuous increase of disease caused by the CRE [3]. Since its emergence in 1996, CRE infections are now endemic in parts of the United States and Europeans countries [1, 4].

However, the prevalence of CRE in the Southeast Asia region was not well documented or under reported [2]. In Malaysia, at the point of this manuscript writing, only two articles regarding CRE have been published, which did not reflect that CRE posed as a major problem [5, 6]. Nevertheless, based on unpublished data, the number of CRE isolated in general and tertiary hospitals is on the rising trend and is alarming. Thus, infection prevention and control (IPC), which refer to a group of interventions including surveillance, standard precautions, hand hygiene and environmental cleaning, plays a vital role in controlling further spread of CRE. Specific IPC measures for MDR-GNB have been derived from current peer-reviewed publications and expert opinion. These measures include surveillance, screening, prevention of transmission, environmental cleaning and many more [7–9].

Management of MDR organism which includes implementation of IPC measures directly lead to the increased use of hospital resources due to extended hospital stays, laboratory tests, physician consultations and costly medications if therapy is needed [10, 11]. Since not all isolation of MDR organisms in clinical specimen warrants treatment, clinical interpretation and selection of screening is crucial, particularly in low-resource countries. Thus, the aim of this study was to determine the number CRE organisms isolated in a university hospital in the east coast region of Malaysia and the common source from which these organisms were isolated.

## Material and methods

This study was a retrospective laboratory-based study conducted from August 2013 to December 2015. All data pertaining to the organisms isolated was retrieved from the WHONET 5.6 databases. In this descriptive study, all CRE isolated within the study period were analyzed including repetitive isolates from the same patients. Enterobacteriaceae was identified based on standard laboratory protocols. All clinical specimens were inoculated on MacConkey agar for isolation of gram-negative bacteria. Isolates were subjected to series of biochemical tests for identification, either manually or using automated identification system, Vitek2® (bioMérieux, France), if necessary. For CRE screening, isolates were tested against imipenem, meropenem and ertapenem antibiotics using disc diffusion method and the MICs were determined by E-test. Antibiotics susceptibility test was performed and interpreted according to the Clinical and Laboratory Standard Institute (CLSI) version 2012 and 2014. MDR is defined as non-susceptibility to at least one agent in three or more antimicrobial categories [12]. CRE was confirmed by exhibition of resistance to imipenem or meropenem and extended spectrum cephalosporin group of antibiotics or positive Modified Hodge test. A one-step PCR assay was used to confirm the genotypic characteristics of the isolates. Since the most common genotypes detected in our local setting were *bla*NDM1 and *bla*IMP4, the isolates in this study were only screened for the presence of *bla*_NDM1_ and *bla*_IMP4_ genes.

## Results

Between 1^st^ August 2013 and 31^st^ December 2015, a total of 8306 Enterobacteriaceae were isolated from 5735 patients. They were isolated from various clinical specimens including screening specimens. Among them, 477 CRE were identified which gave the percentage of 5.74%. Majority of the isolated CRE were *Klebsiella* (408/477, 85.5%), of which *Klebsiella pneumoniae* was the predominant species (388/408,95%). The percentage of other organisms isolated were as shown in Table 1. CRE were isolated mainly from rectal screening, 235 (49.3%), other less common specimens were as shown in Table 2.

**Table 1:** Carbapenem-resistant Entrobacteriaceae isolated from various clinical specimens at Hospital Universiti Sains Malaysia, August 2013 till December 2015

Organism	Number (%)
*Klebsiella*	408 (85.5)
*Escherichia coli*	25 (5.2)
*Enterobacter* sp.	23 (4.8)
*Citrobacter freundii*	20 (4.2)

**Table 2:** Distribution of Carbapenem-resistant Entrobacteriaceae isolated from various clinical specimens at Hospital Universiti Sains Malaysia, August 2013 till December 2015

Type of specimen	Number (%)
Rectal swab	235 (49.3)
Urine	76 (15.9)
Blood	46 (9.6)
Tracheal aspirate	34 (7.1)
Swab	21 (4.4)
Sputum	22 (4.6)
Others	43 (9)

Isolates demonstrated high resistance to the tested antibiotics. Resistance to imipenem and meropenem were 100 and 98.7% respectively. Susceptibility data of ertapenem was incomplete and doripenem was not routinely tested. Susceptibility to colistin/polymyxin and tigecyline was not routinely done unless specifically requested by the treating physicians. Other antimicrobial profiles of the 477 CRE were presented in the Table 3.

**Table 3:** Resistant Pattern of Carbapenem-resistant Entrobacteriaceae isolated from various clinical specimens at Hospital Universiti Sains Malaysia, August 2013 till December 2015

	TZP	CFP	CAZ	CTX	FEP	AMK	GEN	CIP	SXT
Resistance rate (%)	98.3	99.1	98.1	99.4	98.7	79.0	85.5	95.4	91.4

*TZP* piperacillin/tazobactam, *CFP* cefoperazone, *CAZ* ceftazidime, *CTX* cefotaxime, *FEP* cefepime, *AMK* amikacin, *GEN* gentamicin, *CIP* ciprofloxacin, *SXT* trimethoprim-sulphamethoxazole

**Fig. 1:**
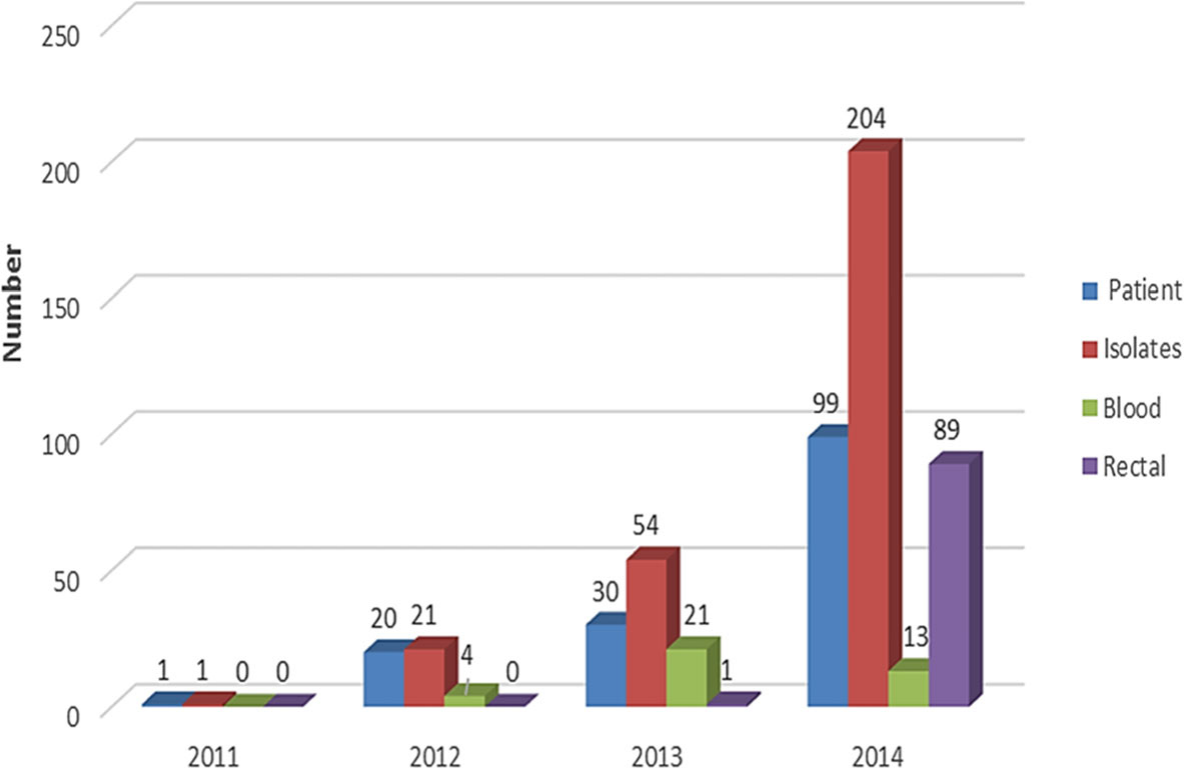
Carbapenem-resistant Enterobacteriacea isolated from various clinical specimens at Hospital Universiti Sains Malaysia, 2011–2014

Separate data analysis has been performed to look at the trend of CRE isolation from 2011 to 2014 since the laboratory documented up surge of the organisms in year 2014. Fifty four CRE were isolated in 2013 compared to 204 CRE in 2014. The surge of CRE was actually due to the increased number of screening specimens. The trend of CRE isolation is shown in Fig. 1. The number of screening specimens was two times higher than the patients themselves. The repetitive specimens were not excluded since screening for CRE was done more than once for some patients.

Out of 477 CRE isolates, only 136 were available for genotyping by PCR. As expected, majority (112/136; 82.4%) of the isolates carry *bla*_NDM1_ gene and one positive for *bla*_IMP4_. The other isolates possibly belong to other genotypes or carry different type of resistance mechanism, which is beyond the scope of this study.

## Discussion

CRE was first identified in North Carolina in 1996 and the incidence rose gradually in USA with few outbreaks. The first outbreak of KPC-producing *K. pneumoniae* outside the USA was reported from Israel. Later, the CRE organisms, mainly KPC-producing *K. pneumoniae* were isolated in South America, European countries and China. The epidemiology of CRE varies according to geographical locations [13, 14]. Their emergence has posed great challenges to the health care facilities due to increased morbidity and mortality. Comparing patients with imipenem and/or meropenem-resistant *K.pneumoniae* infections with carbapenem-susceptible group, 50.0% (10/20) of patients died in the resistant group whereas only 27.5% (11/40) of the sensitive group died during hospitalization [15]. Another study revealed the same findings, in which crude mortality and attributable mortality rate for carbapenem-resistant *K. pneumoniae* bacteraemia were 71.9% (23/32) and 50% respectively. For control subjects, the crude mortality rate was 21.9% (7/32) [16]. To further complicate the issue, effective antibiotics to treat CRE infections are limited, and they are not without unwanted side effects.

The data on CRE prevalence in Southeast Asia is still scarce. Most probably it has not gained much attention in local hospital/institution due to its rare occurrence and under reporting. Furthermore, the prevalence of MDR-GNB varies by countries, institutions and time of the studies [2, 5, 17, 18]. In one study, the authors reviewed the epidemiology of MDR-GNB in Southeast Asia, namely extended spectrum beta-lactamases (ESBL) producers, CRE, MDR-*Acinetobacter* and MDR-*Pseudomonas*. ESBL producers were the main organisms causing infections and were noted to be the major problem, instead of CRE [17]. There were very limited data on CRE, and most studies limitations were related to small numbers of isolates tested in each country. Since ESBL producers were the major MDR-GNB in this region, carbapenem overuse was highly possible or sometimes inevitable. Therefore the emergence of CRE should be anticipated and further studied.

The phenomenon of CRE emergence has also been observed in Malaysia including our local setting. When CRE was first detected in our hospital in 2011, its emergence has alarmed the clinician and infection control team. Thereafter, significant rise in CRE isolation was noted every year. In Asia, during the period of 2001–2012, the resistance rate of Enterobacteriaceae to imipenem and meropenem were 0.8 and 1.0% respectively [2]. From 2000–2003, the resistance rates for both imipenem and meropenem were 0.5%, and the rates increased steadily afterwards. From 2009 to 2012, the resistance rates rose to 1.2 and 1.3% respectively. Though the rate was low, it was on the rising trend [2]. Similar observation was noted in Singapore, where only sporadic cases were detected before 2010. Surveillance was conducted from 2010 to 2013 whereby 400 Enterobacteriaceae isolates with reduced susceptibility to either meropenem or imipenem were analysed. Of the 400 isolates, 227 (56.8%) carried a carbapenemase gene, and *bla*_NDM_ was the most frequently detected (130/227, 57.3%) [18].

It has been well documented that screening for the presence of MDR organism among patients in the healthcare settings is part of the IPC practices worldwide [8, 19, 20]. Rectal screening for CRE was implemented for patient with positive isolation and those who stayed in the vicinity of the index case. It was adopted from the guidelines published by the Ministry of Health of Malaysia and published recommendations [8, 19, 20].

Once positive, patients are isolated and strict IPC measures will be applied and reinforced. However, their effectiveness relies on many factors which are usually difficult to control. Currently, with the uncertainty of world economy and financial status especially in developing countries, routine screening for CRE organisms should be revisited and revised. Screening should be apply for high risk patients and tailored to the specific needs or during outbreaks investigations. Cost pressure for screening is the major determinant in those countries. Estimated laboratory cost for a CRE screening in our local setting can be up to US22 (MYR90) per specimen, which utilized significant amount of laboratory annual budget. Material costs for screening included cost for swabs, culture plates, reagents for bacterial identification and antimicrobial susceptibility. Labor cost must be included as well. Gunther et al. recently reported a cost analysis and possible benefits of a two-tier infection control management strategy for MDR organisms [21]. In the study, high risk patients were screened for the presence of MDR organisms, followed by IPC measures according to the type of MDR isolated. Of 39,551 patients, accounted for 24.5% of total admission during the study period, only 7.8% (3104) were positive for MDR organisms, whereas only 0.3% was positive for XDR organisms including CRE. The study highlighted a low colonization with MDR organisms, even among high risk patients [21]. However, despite the low isolation rate, the cost incurred was not trivial. The mean annual cost for screening was €102,037, of which the main cost factor was allocated for test material. Furthermore, possible transmissions by undetected carriers would have caused additional costs of €613,648.90-€4,974,939.26 [21]. Birgand et al. calculated micro-analysis separately, based on positive or negative cultures. The cost for positive culture for carbapenamase-positive Enterobacteriaceae was €115, including personnel costs for laboratory tests [10]. However, screening cost differs with countries depending on their economic status and financial support. For low-resource setting, taking into consideration all elements of CRE screening, the total expenditure is inevitably tremendous. Normally the allocated funding for medical laboratories is mainly spent on important tests which directly contribute to patients’ management.

The significance and clinical utility of screening results depends on the methods used. Of all methods available, culture is still considered the gold standard. However, given the low sensitivity of the culture method, negative cultures do not truly mean that the patients are free from colonization. Culture detected 77.3% of colonized patients compared to a newer technique, a real-time PCR which was able to detect up 97% of patients [22]. Another study revealed the same findings. Of the 251 consecutive rectal swabs, 30 were PCR positive for one or more carbapenemase genes and only 50% (15/30) of them were culture positive [23]. Poor detection of active cases by conventional culture methods might have contributed to the increasing cases of CRE despite implementation of active screening. Furthermore, as culture results take at least 24–72 h to be available, effective IPC intervention can be delayed.

Effectiveness of the IPC interventions after knowing the colonization status is debatable since many component of the IPC measures need to be monitored. Compliance is one of the factors that need to be emphasized. Huskins et al. conducted a case control study in an intensive care unit, in which colonization status of the patients were ascertained and an additional contact precaution was implemented and compared to the control group. Surprisingly, they found that additional intervention was not effective in reducing the transmission of MDR since many factors influenced the outcome [24]. It has been shown that transmission from patient to patient was mainly via hands of HCWs, although common environmental sources have occasionally been described [9]. Thus, compliance to basic IPC measures (standard precaution) is of utmost importance to control the spread of MDR organisms, regardless of the patient infection/colonization status or types of healthcare settings. The proportion of patients who developed infections after being colonized was less than 10% [25]. However, many guidelines from developed countries with stable economic status recommended active surveillance for patients and contacts to identify unrecognized CRE colonization as clinical cultures alone will identify only a fraction of all patients [8, 19, 20].

Knowing colonization status of a patient is most probably worthwhile for methicillin-resistant *Staphylococcus aureus* (MRSA) since active decolonization can be done according to standard guidelines [8, 19, 20]. On the other hand, management of patients colonized with CRE relies mainly on IPC measures but lacks standardization. There has been wide variation in adoption of screening and infection control interventions for MDR organisms, which reflects the variation of available recommendations and guidelines. Different facilities may have interpreted the guidelines differently and the outcomes may not be the same due to a variety of reasons [26].

Routinely, in patients with positive screening, cohort nursing is implemented with strict adherence to IPC measures. Nevertheless, unless the issue with culture negative screening is resolved, determination of true colonization-free status cannot be made with confidence. In settings with limited funds and resources, routine screening might not be the best measure to control the spread since culture negative patient is unavoidable. Active screening results in significant cost pressures and therefore is not routinely practiced. The best indicator for good control of CRE is most probably to look at the local epidemiology and compliance to basic IPC measures should be emphasized.

## Abbreviations

blaIMP: imipenem metallo-β-lactamases
CLSI: Clinical and Laboratory Standards Institute
CRE: Carbapenem-resistant Enterobacteriaceae
ESBL: Extended spectrum beta-lactamases
MRSA: Methicillin-resistant *Staphylococcus aureus*
NDM: New Delhi metallo-beta-lactamase
PCR: Polymerase chain reaction
